# ROS Gateway: Enhancing ROS Availability across Multiple Network Environments

**DOI:** 10.3390/s24196297

**Published:** 2024-09-29

**Authors:** Byoung-Youl Song, Hoon Choi

**Affiliations:** 1 Electronics and Telecommunication Research Institute, Daejeon 34129, Republic of Korea; sby@etri.re.kr; 2 Department of Artificial Intelligence, Chungnam National University, Daejeon 34134, Republic of Korea

**Keywords:** ROS, on-device robot, middleware, cloud Robotics, gateway, IoRT

## Abstract

As the adoption of large-scale model-based AI grows, the field of robotics is undergoing significant changes. The emergence of cloud robotics, where advanced tasks are offloaded to fog or cloud servers, is gaining attention. However, the widely used Robot Operating System (ROS) does not support communication between robot software across different networks. This paper introduces ROS Gateway, a middleware designed to improve the usability and extend the communication range of ROS in multi-network environments, which is important for processing sensor data in cloud robotics. We detail its structure, protocols, and algorithms, highlighting improvements over traditional ROS configurations. The ROS Gateway efficiently handles high-volume data from advanced sensors such as depth cameras and LiDAR, ensuring reliable transmission. Based on the rosbridge protocol and implemented in Python 3, ROS Gateway is compatible with rosbridge-based tools and runs on both x86 and ARM-based Linux environments. Our experiments show that the ROS Gateway significantly improves performance metrics such as topic rate and delay compared to standard ROS setups. We also provide predictive formulas for topic receive rates to guide the design and deployment of robotic applications using ROS Gateway, supporting performance estimation and system optimization. These enhancements are essential for developing responsive and intelligent robotic systems in dynamic environments.

## 1. Introduction

The convergence of technological advancements in sensor and actuator technology, the diversification of robotic components, and notable enhancements in computing power have broadened the applicability and scope of robots across a diverse array of industries [1]. Sensors have become more accurate, affordable, and diverse, thereby enabling robots to perform complex tasks with greater precision and adaptability in dynamic environments [2]. Similarly, actuators, which control movement and operation, have undergone significant advancement, resulting in improved control, efficiency, and reliability. Furthermore, advancements in artificial intelligence (AI) are transforming robots by enabling autonomous decision-making, environmental learning, and enhanced performance [3]. These technological developments have established robots as indispensable assets in a multitude of sectors, including the Internet of Things (IoT) [4,5], manufacturing [6], agriculture [7,8], logistics [9], healthcare [10,11], and smart cities [12,13].

As robotic systems evolve, the necessity for uninterrupted communication and connectivity between these systems and broader infrastructures becomes increasingly apparent. The rapid advancement of communications technology, in conjunction with cloud computing, is exerting a considerable influence on the field of robotics [14,15,16,17,18]. In particular, cloud computing provides enhanced processing power, scalability, and storage capacity, thereby enabling robots to offload intensive tasks such as AI computations to the cloud. This offloading of processing tasks reduces the burden on local hardware and enables the integration of robots into larger, interconnected systems that operate across multiple locations. In domains such as the Internet of Things (IoT), manufacturing, and logistics, it is imperative that robots are able to communicate with one another, share data in real-time, and integrate seamlessly with external systems in order to optimize performance.

The Robot Operating System (ROS) [19] has gained considerable traction as a framework for robotics research and development, largely due to its modularity, ease of use, and community-driven evolution. Despite its extensive adoption, ROS encounters considerable obstacles, particularly with regard to its capacity to connect to cloud-based infrastructure. As a middleware solution for the transmission of sensor data, ROS typically assumes distributed computing over local area networks (LANs). In ROS1 [20], peer-to-peer communication is dependent on a centralized system that is only effective when the peers are situated within the same network environment. This may include the sharing of public Internet Protocol (IP) addresses or the existence of the same subnet. In contrast, ROS2 [21,22] is operational only in networks that support multicast for peer discovery. These constraints restrict the functionality of ROS in environments that necessitate remote communication over expansive networks or networks with limited bandwidth, such as those employed in cloud or fog robotics applications [23].

It is therefore imperative to develop a robot communication middleware that can facilitate seamless connections between robots and the cloud. A number of researchers have put forth proposals aimed at enhancing communication, optimizing data transfer, and guaranteeing secure connectivity between robotic systems and cloud infrastructure. Nevertheless, although these solutions are effective for particular tasks, they exhibit shortcomings in terms of flexibility, which is essential for addressing the diverse needs of robotic and cloud computing environments. For instance, it has been observed that rosbridge [24]-based solutions [25,26,27], which are frequently employed within the ROS community, often exhibit an elevated latency when transmitting large data volumes [28]. Moreover, these solutions are designed exclusively for the server role, which restricts their applicability to mobile devices with dynamically allocated IP addresses. In contrast, solutions based on virtual private networks (VPNs) [29,30,31] present a more complex system setup, exhibit high latency, and are ineffective at transmitting high data volumes due to inherent size limitations. Moreover, the lossy video compression techniques employed for high-speed image transmission in VPN-based solutions are unsuitable for transmitting large volumes of non-video data, such as point clouds. In contrast, Message Queuing Telemetry Transport (MQTT) [32] and ZeroMQ 4 [33]-based solutions [28,34,35,36] necessitate the development of custom software for each application and support solely ROS’s topic communication method, which impedes the integration of diverse ROS-based robotic components. This issue will be explored in greater depth in Section 2.2.

In response to these challenges, we propose ROS Gateway, a novel ROS-based cloud middleware designed to extend ROS functionality into the cloud in a seamless and non-invasive manner. The objective of ROS Gateway is to provide a robust communication infrastructure that facilitates the sharing of sensor data, enhances scalability, and enables integration with cloud services during operation. In contrast to preceding middleware solutions, ROS Gateway prioritizes flexibility, thereby enabling robots to capitalize on cloud-based resources in a more efficacious manner while maintaining uninterrupted communication with connected systems. By addressing the limitations of ROS connectivity, ROS Gateway aims to facilitate the full potential of cloud computing in robotics, thereby contributing to the continued advancement of the field.

This study makes the following contributions:

- This paper presents ROS Gateway, a middleware designed to accelerate ROS topic communication for the collection and processing of sensor data from robotic systems. It provides a detailed account of the Gateway’s architectural design, communication protocols, and underlying algorithms. ROS Gateway facilitates the implementation of ROS functionality across a range of networks through the use of a straightforward JavaScript Object Notation (JSON) [37]-based configuration.
- In an experimental setting where substantial quantities of sensor data, exceeding hundreds of kilobytes and generated at rates above 10 Hz, were produced, experiments were conducted to evaluate the transmission performance based on different robot application configurations.
- Formulas for calculating the predicted ROS topic reception rates based on the configuration settings of the robot applications were developed. The formulas provide an approximation of the predicted performance in both cases, that is, when using only ROS and when using ROS Gateway.

The remainder of this paper is organized as follows. Section 2 discusses the motivation behind this research and related work, highlighting the main differences between the proposed middleware and state-of-the-art solutions. Section 3 describes the architecture, protocols, specifications, and algorithms of the proposed middleware. Section 4 presents a comparative study of the proposed middleware against alternative solutions under various experimental performance configurations. Finally, the paper concludes with a discussion of future work.

## 2. Background

### 2.1. Motivation

**Figure 1:**
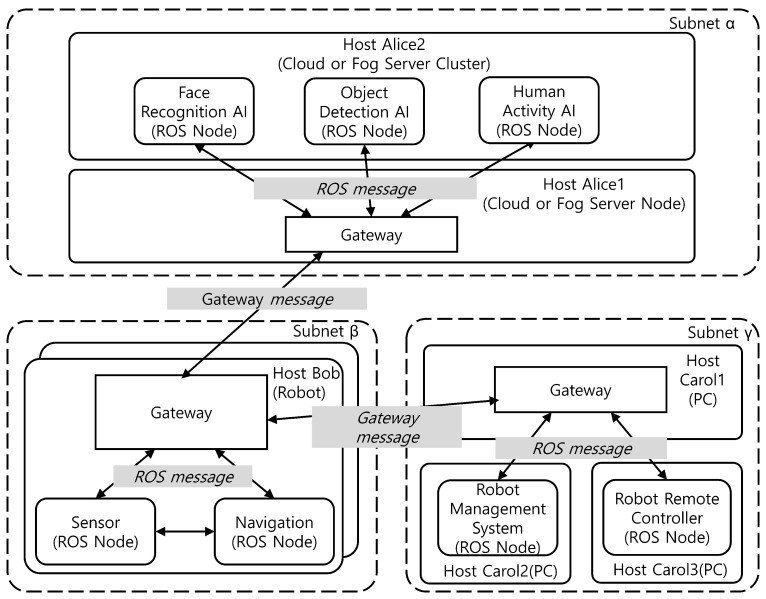
Example of a ROS-Based robotic application in cloud or fog Configuration. Subnet α is a subnet to which multiple hosts that constitute cloud or fog computing are connected. Subnet β is a subnet to which robots are connected, or a local host network within the robot. Subnet γ is a subnet to which a control system consisting of multiple hosts in a remote location is connected, though not a subnet in which robots are included.

Figure 1 illustrates a typical example of a robotics application utilizing ROS in a cloud or fog computing configuration. In this configuration, the robot’s sensor data is processed by a control module integrated into the robot itself, and transmitted to a remote server for further processing and utilization by AI services. The robot management and control system, situated on a separate network, is capable of receiving and monitoring the robot’s sensor and status data, as well as remotely controlling the robot in the event of anomalies. In such a scenario, the robot is typically assigned a dynamic IP address, while the cloud server or cluster and the control computer are assigned static IP addresses. From this analysis, we have identified several additional middleware requirements that can improve the usability of ROS, as summarized in Table 1.

### 2.2. Related Works

Rosbridge [24] was developed with the objective of addressing the challenge of interoperability by enabling communication between ROS and non-ROS systems. It is a widely utilized tool within the robotics community that employs ROS due to its compatibility with the communication characteristics of ROS and its capacity to integrate with web-based ROS tools. In their respective studies, Galarza et al. [25] and Shamaine et al. [26] proposed a system for remotely controlling robots through virtual reality. In their study, robots based on the Robot Operating System (ROS) are connected to Unity via rosbridge, thereby enabling manipulation of these robots in a virtual environment. Wang et al. [27] presented a general-purpose ROS robot live interaction platform, designated TeleRobot, which provides a range of functions, including multi-angle robot live streaming, remote interaction with robots, and online discussions. In their paper, the authors indicated that they utilized rosbridge to transmit and receive remote control commands and feedback data from the robot, thereby ensuring compatibility with a wider range of robots within the TeleRobot platform. Despite its numerous applications, rosbridge has been observed to exhibit communication performance issues in data-intensive contexts, particularly in the retrieval of video and point cloud data [28]. Its functionality is largely limited to the retrieval of data from ROS on remote systems and the transmission of basic control commands.

Chen et al. [29] presented FogROS, which extends ROS executable scripts to enable the deployment of ROS1 nodes in a cloud environment. This allows robots to offload heavy computations to cloud services such as Amazon Web Services (AWS). Subsequently, Ichnowski et al. [30] presented FogROS2, an open-source platform that integrates cloud and fog computing with ROS2. This allows robots to utilize cloud resources for computationally intensive operations without requiring modifications to existing code. Meanwhile, Damigos et al. [31] proposed a novel architectural approach that integrates ROS and 5G for the offloading of unmanned aerial vehicle (UAV) control to edge servers, thereby ensuring low-latency and reliable communication. The researchers utilized a virtual private network (VPN) to facilitate communication between the robot and the edge server, demonstrating that this approach is secure but results in increased system latency due to transmission overhead. These platforms provide scalable and flexible computing solutions that facilitate the configuration of cloud-based resources based on a VPN and enhance the capabilities of robotic systems. However, inherent limitations of VPN approaches include the inability to forward ROS traffic generated from external computers on the same network without VPN installation and difficulty in forwarding large data types, such as images and point clouds. FogROS2 employed a distinctively configured streaming server for the transmission of video. However, due to its use of video-based lossy compression, its applicability to the transmission of high-volume non-video data is limited.

Coronado et al. [28] identified deficiencies in the communication performance of rosbridge, which facilitates communication between ROS and devices or programming languages not supported by ROS, and is convenient for programming sensor information. As an alternative, they proposed NEP+, an IoT framework based on ZeroMQ [33] and ROS2, which enables seamless integration of human–machine systems across platforms. The proposed framework is designed to enhance human–robot collaboration by providing low latency and robust interoperability. Furthermore, it has the potential to be extended to other platforms, such as Unreal Engine and Nvidia Omniverse. It is evident that the authors’ findings present a viable alternative for the implementation of cloud robotics middleware. Nevertheless, it proved difficult to utilize as a middleware solution that would adequately address the aforementioned challenges.

Lertyosbordin et al. [34] presented a framework that integrates ROS and Message Queuing Telemetry Transport (MQTT) [32] to enable remote robot command and operation from Internet-connected devices. Moreover, Lampe et al. [35] put forth the concept of RobotKube, a system that orchestrates containerized microservices in large-scale multi-robot systems using Kubernetes and ROS. The methodology is proposed for the implementation of cooperative intelligent transportation systems (C-ITS), utilizing ROS topic data for the exchange of information between vehicles and the cloud through MQTT. Moon et al. [36] proposed the Edge-Driving Robotics Platform (EDRP) for autonomous driving, which is based on a microservice architectural framework. Furthermore, they proposed the Local Dynamic Map Platform (LDMP), which facilitates the real-time sharing of dynamic object information. In their paper, the authors utilized an MQTT broker to relay ROS topic messages from autonomous robots to EDRP within a 5G mobile edge computing system. MQTT-based platforms offer a number of advantages, including a minimal overhead, publish-subscribe protocols that align with the topic concept of ROS, and low power characteristics. However, as previously suggested, these implementations are designed for specific applications and do not support the communication characteristics of ROS beyond the topic method.

The aforementioned studies are insufficient for addressing the presented challenges. This is due to the fact that they exhibit shortcomings such as limited scalability, suboptimal communication performance, and an inability to integrate a diverse range of robot components utilized in ROS-based systems, thereby hindering the ability to accommodate the heterogeneous robot and cloud computing environments. Table 2 provides a comparative analysis between the proposed system and previous studies.

The following is a description of each of the features presented in Table 2.

- ROS Topic: Indicates whether the system supports ROS topic-based interaction between the robot and the cloud.
- ROS Service: Confirms whether the system supports ROS service-based interactions between the robot and the cloud.
- ROS Action: Specifies whether the system allows ROS action-based interactions between the robot and the cloud.
- Provisioning: Determines whether the system can provision ROS interaction from inside the robot to the cloud.
- Cloud to Robot: Confirms whether the system can transfer ROS interaction from the cloud to the robot.
- Non-invasive: Indicates if the system can integrate with existing systems without requiring major modifications or disruptions.
- General Purpose: Evaluates whether further development is necessary, depending on the specific application.
- NAT/Firewall: Confirms whether the system can operate within a NAT or firewall environment.
- Web Compatibility: Specifies whether the system is compatible with web-based ROS tools.
- Cloud Protocol: Identifies whether the system uses cloud-friendly protocols, such as HTTP.
- Multi-Host ROS: Determines whether the system is easy to use for multi-host ROS interaction across different networks.
- Large Data: Confirms whether the system supports large-scale data interaction.

## 3. Proposed Scheme

This section provides a description of the ROS Gateway’s architecture, protocols, and algorithms. Additionally, the Appendix A presents a logic model of how each task operates in conjunction with each algorithm, illustrated through a sequence diagram.

### 3.1. Architecture

**Figure 2:**
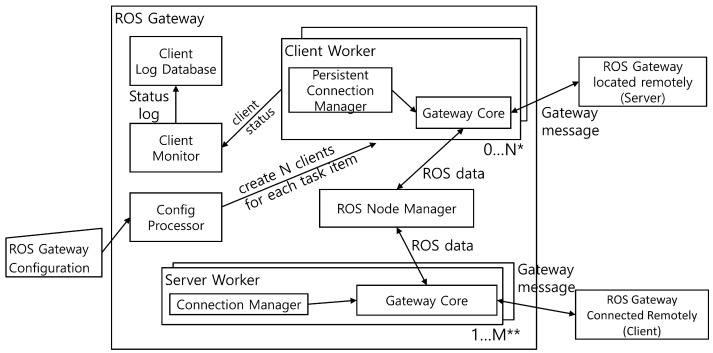
The Gateway architecture. * The Client Worker is activated when a configuration that enables connectivity to a gateway in a different network is applied. ** Server Workers are initially created with a single process for receiving commands from external sources. Subsequently, a new process is assigned based on the client that establishes a connection.

The proposed system comprises a number of key components, designed with the objective of facilitating communication between ROS nodes across different hosts. Figure 2 provides an overview of the system architecture at a high level of abstraction. As illustrated in the figure, the Server Workers are responsible for managing incoming connection requests from clients, while the Client Workers establish connections with Gateways on other hosts. The Client Monitor is responsible for monitoring the operational status of the client role Gateway, while the Client Log Database records runtime configuration settings. This enables the Gateway to reinstate previous configurations upon restart. The Config Processor is responsible for processing the configuration JSON transmitted to the Gateway, while the ROS Node Manager facilitates communication with ROS nodes. The Server Workers and Client Workers interact with ROS nodes via the ROS Node Manager, processing received Gateway messages or executing operations defined by the Configuration JSON using the Gateway Core. In order to optimize system performance, the Gateway proactively creates and manages a pool of Server Worker processes, with the pool size (M) defined by the user. This pool is responsible for the management of Server Workers corresponding to client Gateways that are connected at runtime. The Connection Manager, which is embedded within each Server Worker, logs pertinent client connection information, while the Persistent Connection Manager, which is situated within the Client Worker, maintains communication with the Gateway on other computers in order to perform the operations that have been configured, provided that the transmitted Configuration JSON is not revoked. Section 3.2 provides a comprehensive analysis of the Gateway protocol messages. Section 3.3 presents a detailed examination of the format of the Gateway configuration specification. Section 3.4 outlines the operations performed by the Gateway based on the configuration specification and protocol messages.

### 3.2. Gateway Protocol Messages

The Gateway employs the WebSocket [41] communication methodology and adheres to the specifications set forth in rosbridge protocol version 2.0 [42], thereby ensuring compatibility with existing ROS web-based tools [38,39,40]. To enhance the efficiency of message transmission, the Gateway now offers the option of compression for both outgoing and incoming messages. Furthermore, the Gateway introduces the ability to transmit compressed binary data in the zip and rawzip options, providing an alternative to the existing Portable Network Graphics (PNG) compression method. The *PNG* option of rosbridge resulted in reduced performance due to the necessity of Base64 [43] encoding for the transmission of compressed binary data.

Table 3 provides a brief description of the Gateway protocol messages, which have been designed to extend the rosbridge protocol [42]. The structure of each message is based on a JSON format and is transformed into either a binary array encoded with Concise Binary Object Representation (CBOR) [44] or a JSON message in text format, depending on the values specified in the compression field prior to transmission. The op field is a mandatory component that is utilized to specify the message’s purpose, whereas the id field is employed to uniquely identify the message and prevent duplication. Furthermore, the id field enables the matching of response messages to their corresponding request messages, which is particularly advantageous when transmitting ROS service and ROS action [45] data.

The rows designated as (a), (b), and (c) in Table 3 pertain to ROS topic-related messages. The topic field denotes the name of the ROS Topic, while the type field indicates the data type associated with the topic. The field designated as msg contains data pertaining to the ROS topic, which is presented in either a JSON string or a binary array, contingent upon the selected compression settings. Messages belonging to categories (d) through (f) pertain to ROS services. The service field specifies the name of the ROS service, whereas the type field defines the type of the ROS service in question. The field designated as args contains the input arguments, which are encoded as either a JSON string or a binary array.

Table 3 also includes entries (g) to (j), which pertain to ROS action-related messages. In these messages, the action field denotes the designation of the ROS action, whereas the type and action_type fields specify the type of the ROS action. In (h), the args field is utilized to delineate the argument value for the ROS action goal, which is encoded in either a JSON string or a binary array. The feedback field in (h) indicates whether the recipient should receive feedback regarding the action. The intermediate result values for ongoing ROS actions are indicated in (i), while the final result values upon goal completion are presented in (j). The result field in (j) indicates the outcome of the action, with a value of true signifying success.

With regard to the available compression options, the zip option employs CBOR to serialize ROS data prior to applying a compression algorithm, whereas the rawzip option directly compresses raw ROS data using the same algorithm. These compression options are designed to target actual ROS data values. They can be applied to the msg field in protocol message (b), the args fields in protocol messages (e) and (h), and the values fields in protocol messages (f), (i), and (j). In order to apply the cor, cbor-raw, zip, or rawzip compression options to a protocol message, it is necessary to first convert the entire JSON-formatted message into a binary array through CBOR encoding prior to transmission.

### 3.3. Gateway Configuration Specification

The Gateway configuration allows for the creation of virtual ROS networks that can operate over a variety of network infrastructures, thereby facilitating seamless collaboration between ROS programs in disparate environments. This configuration, expressed in JSON format, can be applied dynamically in one of two ways: either via a startup file passed as an argument to the Gateway at runtime, or through the Gateway’s web interface. Furthermore, the configuration file can consolidate the connectivity and operational instructions for Gateways running on disparate hosts across heterogeneous networks. This flexibility permits the configuration of a ROS virtual network that is optimized for the requirements of complex robotics applications that involve the integration of multiple cloud or fog servers and robots.

In the Gateway configuration, the commands that a Gateway should execute are defined as a collection of JSON data, with each segment corresponding to a specific target Gateway. Each collection of operations is associated with a distinct target Gateway address. These commands include the forwarding of ROS topics to a different network (referred to in the protocol as a publish operation) and the reverse forwarding of ROS topics to retrieve those distributed solely on other networks (referred to as a subscribe operation). Moreover, the configuration includes commands to expose ROS services and actions that are only available on the local subnet, as well as to prepare the Gateway to make ROS services and actions available locally that were previously restricted to remote networks. The subnet is connected to remote networks (referred to as expose-service and expose-action operations), while additional commands prepare the Gateway to make remote ROS services and actions available on the local subnet (referred to as reserve-service and reserve-action operations). Each operation command is comprised of three distinct fields. The name field identifies the target ROS topic, service, or action; the type field specifies the data type; and the compression field describes the encoding and compression techniques to be applied to the message.

The JSON configuration presented in Listing 1 illustrates the operational command settings for the tasks to be performed by the Gateway on Host Bob in order to configure the scenario depicted in Figure 1. The file delineates the operational parameters of the Gateway, which facilitate the extension of ROS-based interaction by establishing connections with Gateways on two additional networks. The initial directives, delineated in lines 2 through 7, facilitate the exposure of the ROS service (pause_robot) and ROS action (navigate_to_ros) provided by the robot to the Gateway on a subnet operating on Host Carol1. Additionally, the ROS service (controller_start) is scheduled to signal the startup of robot control. Consequently, the Robot Management System, which is running on Host Carol2 within the same subnet, is now able to position the robot and transmit commands to it. Furthermore, the Robot Remote Control node on Host Carol3 is able to invoke the emergency stop service (pause_robot) by utilizing the ROS service (controller_start) that was previously called on Host Bob during robot operation.

Subsequently, directives located in lines 8 through 14 utilize the cbor-raw compression option to encapsulate the image topics (image_raw) distributed by Host Bob’s Sensor node and forward them to the subnet α via Host Alice1’s Gateway. Furthermore, the directives include the reverse-forwarding of topics such as face_recog_result, object_detect_result, and human_act_recog_result, which are generated by AI services running on a cloud (or fog) server. This enables the robot’s internal environment to access these topics, allowing the robot to perform intelligent tasks, including obstacle avoidance, human tracking, and remote control. This is achieved by leveraging high-level AI services hosted on high-performance servers.


**Listing 1.** An example JSON configuration for the Gateway.1
[{
2 "address":"ws://*<address of Host Carol1>* ",3 "expose-service":[{"name":"/pause_robot","type":"std_srvs/srv/Empty"}],4 "expose-action":[{"name":"/navigate_to_pose",5       "type":"nav2_msgs/action/NavigateToPose"}],6 "reserve-service":[{"name":"/controller_start","type":"std_srvs/srv/SetBool"}]7
},
8
{
9 "address":"ws://*<address of Host Alice1>* ",10 "publish":[{"name":"/image_raw","type":"sensor_msgs/msg/Image",11       "compression":"cbor-raw"}}],12 "subscribe":[13       {"name":"/face_recog_result","type":"std_msgs/msg/String"},14       {"name":"/object_detect_result","type":"std_msgs/msg/String"},15       {"name":"/human_act_recog_result","type":"std_msgs/msg/String"}]16
}]


### 3.4. Gateway Algorithms to Enhance Availability of the ROS

This section outlines the algorithms and processes utilized by the Gateway to facilitate the interconnection of ROS topics, services, and action nodes that are distributed across multiple separate networks. These algorithms encompass a description of the Bring Up procedure for initiating the Gateway operation and procedures for making ROS topics, services, and actions available through the Gateway between nodes on separate networks. With these algorithms, the Gateway establishes a virtual wide-area ROS network, thereby ensuring seamless and efficient interaction between ROS modules.

#### 3.4.1. BringUp Task: Gateway Task Startup Procedure

The Algorithm 1 presented here outlines the BringUp procedure, which initiates a worker process responsible for executing the Gateway operations associated with ROS topics, services, and actions. This process is predicated upon the configuration values supplied to the Gateway. Each task is characterized by a configuration c, which encompasses four constituent elements:

- o: The operation directive (e.g., publish, subscribe, expose-service, reserve-service, expose-action, reserve-action, and see Section 3.3 for detailed descriptions).
- u: The endpoint address of the target Gateway that the Gateway will contact in order to perform the task.
- n: The identifier of the ROS topic, service, or action, as determined by ROS naming conventions.
- t: The data type associated with the topic, service, or action, as applicable.

The OpClassOf function categorizes the operation type of each task into one of three classifications: topic, service, or action. The Hash function is employed to generate a unique key for each task configuration, which is subsequently utilized to mitigate bridging loops wherein the client-role Gateway and the server-role Gateway perpetually exchange identical topics. The CreateClientTaskProcess function generates a client task process customized to the operational directive, contingent upon the operation type of the task. The following task types may be instantiated:

- TopicTask: Responsible for managing ROS topics (refer to Algorithm 2);
- ServiceTask: Responsible for managing ROS services (refer to Algorithm 3);
- ActionTask: Responsible for managing ROS actions (refer to Algorithm 4).

The CreateMessageEndpointProcess function is responsible for coordinating connections originating from other Gateways and, in turn, initiating the creation of MessageEndpoint functions in response to the operation directives solicited from other Gateways. These tasks are carried out in accordance with Algorithms 2–4. This algorithm effectively oversees and synchronizes ROS topics, services, and actions across the Gateways, thereby establishing a virtual wide-area ROS network interconnected through the Gateways. This facilitates seamless communication and collaboration among ROS modules.
**Algorithm 1.** Gateway Task BringUp
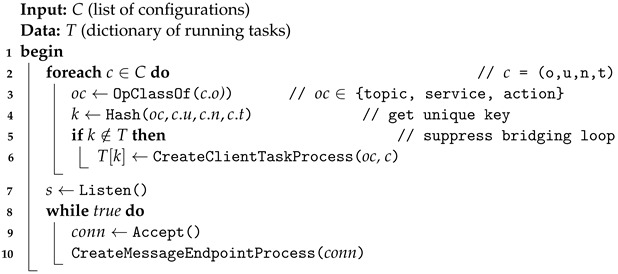


**Algorithm 2.** Gateway Topic Task Process

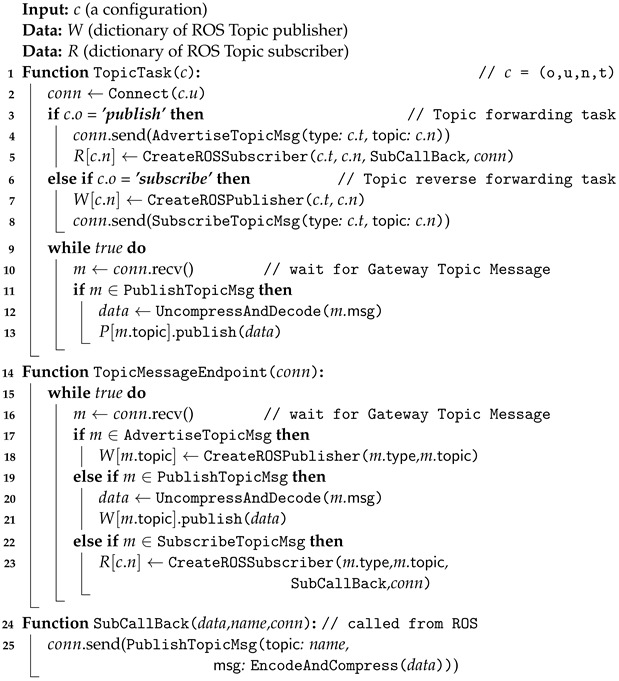



**Algorithm 3.** Gateway Service Task Process

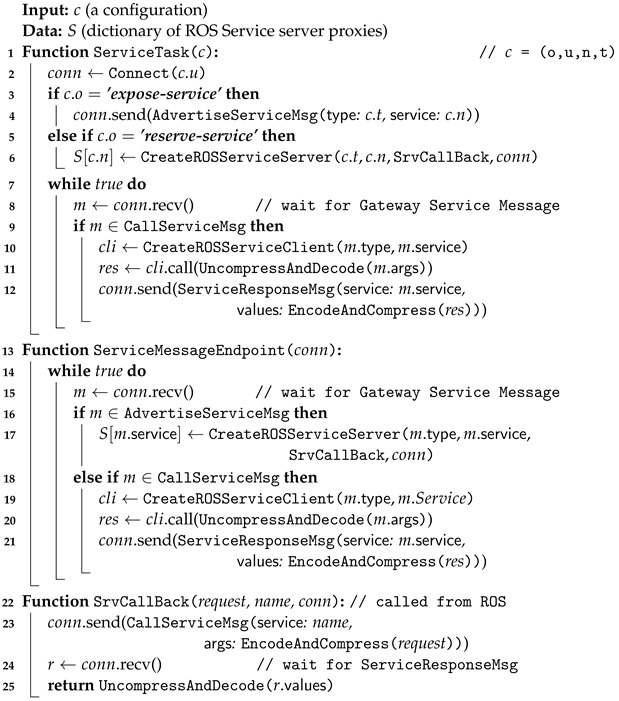



**Algorithm 4.** Gateway Action Task Process

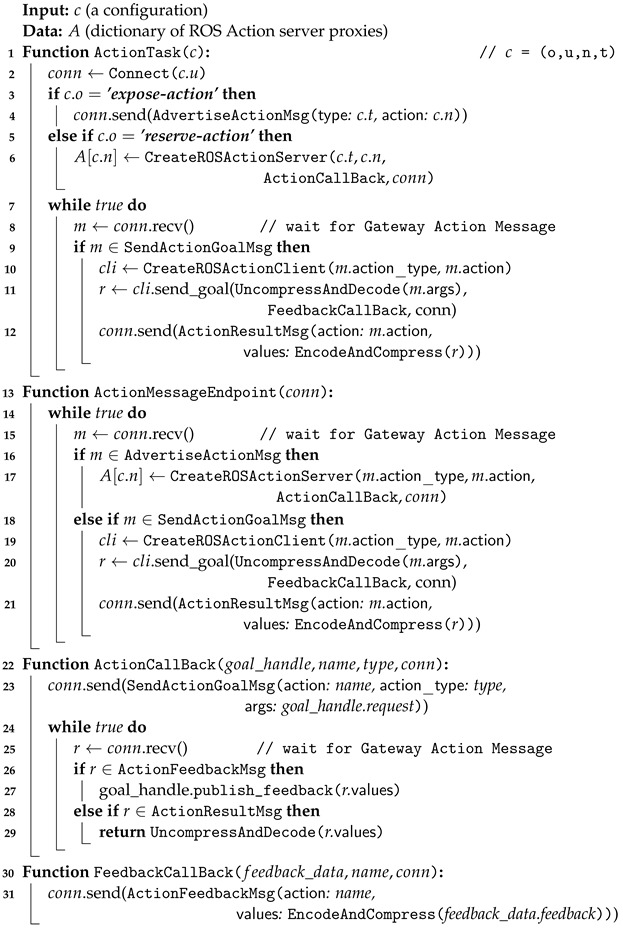



#### 3.4.2. Topic Task: ROS Topic Communication across Different Networks

The algorithm presented in Algorithm 2, entitled “Gateway Topic Task Process”, describes the process by which a Gateway connects ROS topic publishers and ROS topic subscribers on different networks. This algorithm is required to facilitate ROS topic communication between ROS nodes on networks with different address ranges (i.e., separate networks). The algorithm accepts a configuration parameter, designated *c*, which represents task details, including the operation (*o*), Uniform Resource Identifier (URI) (*u*), topic name (*n*), and topic type (*t*). Additionally, dictionaries *W* and *R* are employed to store the ROS topic publishers and ROS topic subscribers, respectively.

**Figure A1:**
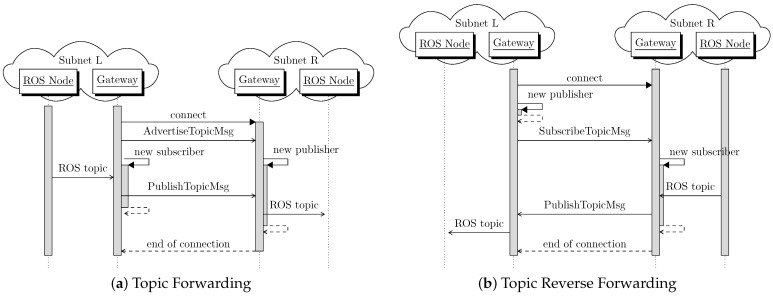
Topic task sequence diagram.

The TopicTask function is responsible for executing the topic operation in accordance with the specifications set forth in the configuration. In the event that the operation directive is publish, a connection is established and a topic forwarding message (i.e., AdvertiseTopicMsg) is sent to the connected Gateway. Conversely, in the case of a subscribe operation, the function creates a ROS topic publisher and sends a topic reverse forwarding message (i.e., SubscribeTopicMsg) to the connected Gateway. In both cases, the function continuously monitors incoming messages and forwards them in accordance with the specified operation. The TopicEndpoint function serves as the endpoint for the processing of topic-related messages received from the Gateway. The function monitors incoming messages and executes the requisite tasks based on their type. In the event that the received message is a topic forwarding message (i.e., AdvertiseTopicMsg), the TopicEndpoint function initiates the creation of a publisher for the topic message to be forwarded. In the event that the received message is a topic data message (i.e., PublishTopicMsg), the TopicEndpoint function decodes the data contained in the msg of the message and subsequently publishes it through the ROS topic publisher. Similarly, if the message is a topic reverse forwarding message (i.e., SubscribeTopicMsg), the TopicEndpoint function establishes a subscriber for the requested topic. Furthermore, the algorithm defines a callback function, SubCallBack, for handling incoming messages from ROS subscribers. Upon receipt of ROS data, this function compresses and encodes it, subsequently passing it to the target Gateway for publication. In essence, this algorithm provides an organized approach to managing ROS topics within the Gateway, thereby enabling seamless communication between nodes on separate networks. A sequence diagram illustrating the operation of this algorithm, triggered by the BringUp Task Algorithm 1, is provided in Figure A1 of Appendix A.

#### 3.4.3. Service Task: Enhancing ROS Service Interoperability across Networks

The algorithm delineated in Algorithm 3 provides a comprehensive explanation of the Gateway Service task process, which has been designed to facilitate enhanced interaction between ROS service servers and clients operating across disparate networks. This process encapsulates the service logic necessary for the dynamic handling of ROS service requests and for extending ROS service accessibility across multiple networks with varying address ranges through a standardized communication protocol. The fundamental elements of this algorithm encompass the initialization of service task functions, message processing, and service invocation. These elements are elucidated through a series of function definitions and message processing logic.

The ServiceTask function establishes initial connections and determines whether to expose or reserve ROS services based on the configuration parameter *c*. In response to the expose-service directive, the Gateway establishes a connection with the message endpoint of the target Gateway, which is operating within a different network. Subsequently, the Gateway transmits a service advertisement message (i.e., AdvertiseServiceMsg) to the target Gateway, thereby exposing the ROS service. In contrast, when the reserve-service directive is employed, the Gateway establishes an ROS service server with specified service types, service names, and callback functions, subsequently registering it in the service server dictionary *S*. The iterative loops within the ServiceTask function are designed to facilitate the effective handling of service call messages received from other Gateways requesting exposed services. The ServiceEndpoint function is responsible for monitoring and responding to incoming messages from external Gateways. Upon receipt of a service advertisement message, the system instantiates and registers an ROS service server. Upon receipt of a service call message (i.e., CallServiceMsg), the system creates an ROS service client, decompresses and decodes the arguments from the message, and invokes the corresponding ROS service. In response to an ROS service call, the data received from the ROS node is encoded, compressed, and then transmitted back to the client in a Gateway service response message (i.e., ServiceResponseMsg). The SrvCallBack function serves as a service callback for the ROS service server and is designed to encapsulate the logic required to enable the fulfillment of service requests from ROS nodes on a separate network via the Gateway. Upon invocation of an ROS service by an ROS node, the request data is routed to SrvCallBack. The ROS data is encoded, compressed, and included in a Gateway service call message that is transmitted to the connected Gateway. Subsequently, it awaits the service response message, decodes it, and returns the result. This function facilitates the forwarding of ROS service calls from ROS nodes to other Gateways.

**Figure A2:**
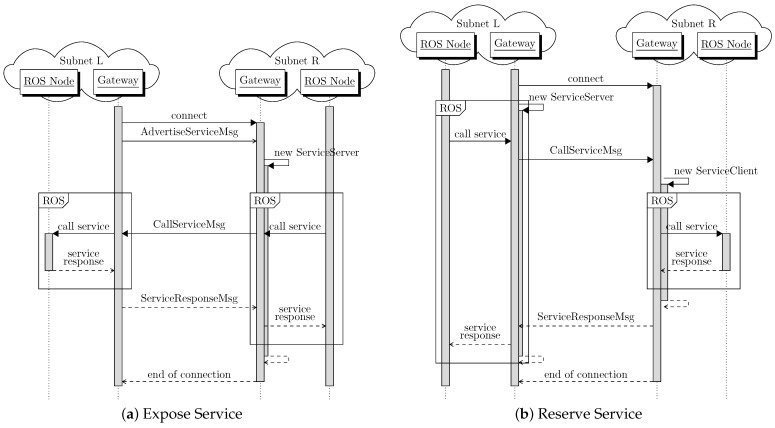
Service task sequence diagram.

The goal of this algorithm is to extend the accessibility of ROS services to devices without a fixed address. In addition, it facilitates interoperability between ROS service servers and clients within distributed networks connected through ROS Gateways in the cloud. The sequence diagram in Figure A2 in Appendix A illustrates the behavior of the Gateway service task, which supports provisioning ROS services to the cloud or late binding to ROS services provided by the cloud according to the algorithm.

#### 3.4.4. Action Task: Multi-Network Communication Methods for ROS Actions

The algorithm presented in Algorithm 4 establishes a connection between ROS action servers and action clients on disparate networks. This enables the ROS action functionality, as provided by ROS, to operate across multiple distinct networks. The algorithm comprises a number of pivotal functions and a set of message processing logic, which have been devised with the objective of facilitating the multi-network interaction of ROS actions.

The ActionTask function establishes the initial connection for other target Gateways and determines whether to expose or reserve the action based on the configuration parameter *c*. In response to the expose-service directive, the function sends an action advertisement message (i.e., AdvertiseActionMsg) to the connected Gateway. Subsequently, it awaits the receipt of messages from the connected Gateway. In the event that the received message is a request for a ROS action (i.e., SendActionGoalMsg), it creates a ROS action client to invoke the ROS action. This is achieved by configuring the ROS action arguments from the received message. Subsequently, upon receipt of the result from the ROS action server running on another ROS node in the local subnet, the message is configured for transmission of ROS action results (i.e., ActionResultMsg) in the Gateway protocol and forwarded to the Gateway that previously sent the action execution request message.

Upon receipt of the action advertisement message, the ActionMessageEndpoint identifies the action type and action name from the incoming message and initiates the creation of an ROS action server with action callbacks configured to facilitate the actual action service. In the event that the ROS action is invoked from another ROS node that is on the same network as the Gateway in question, the request data is transmitted to the callback function of the ROS action server that has been created here. The ActionCallBack function generates a request message for a Gateway ROS action (i.e., SendActionGoalMsg) based on the received ROS action data (i.e., goal_handle.request) and transmits it to the connected Gateway. Subsequently, it remains in a state of continuous anticipation for an action result message from the connected Gateway. Concurrently, upon receipt of action feedback messages, it persists in disseminating feedback (i.e., publish_feedback) to the ROS node that initially requested the ROS action.

In the event that the subnet containing the Gateway running the ActionTask lacks a corresponding ROS action server and the ROS action is being served from a separate network, the *reserve-action* directive may be employed. To facilitate the configuration of action reservations, the ActionTask function establishes a ROS action server with the objective of receiving ROS action data from ROS nodes within the same address range as the Gateway. The ROS action server is created with four arguments: an action type, an action name, an action callback, and the target Gateway connection handle. Subsequently, the logic for processing the action reservation is executed solely within the ActionCallBack and FeedbackCallBack functions. The subsequent step is to transfer the action data from the ROS node to the connected Gateway and relay the received Gateway action messages (i.e., ActionFeedbackMsg, ActionResultMsg) to the ROS node. The aforementioned logic is employed once more in the context of ROS action reservation, given that the ROS action exposure task and message passing direction are in opposition, yet the processing process remains identical.

**Figure A3:**
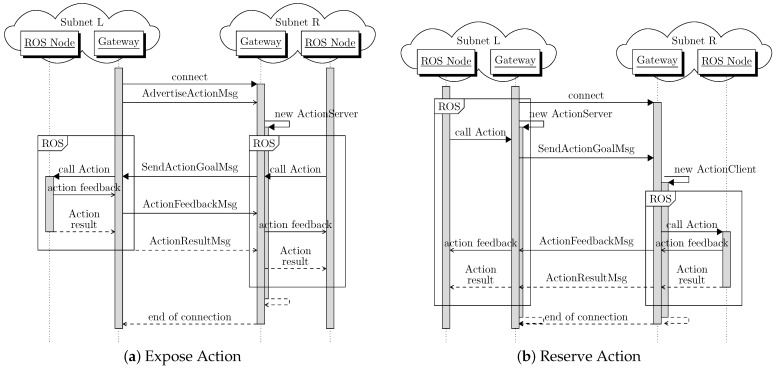
Action task sequence diagram.

This methodology has the potential to enhance system stability and performance when configuring robot applications. It enables external control of ROS action functions within the robot and facilitates the configuration of robot applications using the high-performance action function provided by a cloud server with advanced computing capabilities. The sequence diagram in Figure A3 in Appendix A illustrates how the Gateway extends ROS actions across the network based on this algorithm.

### 3.5. Gateway Features Overview

The principal characteristics of the Gateway, as implemented through the proposed scheme, are as follows:

- Combines both client and server functionalities into a single agent.
- Supports the rosbridge protocol, enabling integration with rosbridge-based web tools.
- Includes protocol modifications to address performance issues without compromising webtool compatibility.
- Allows interaction with ROS web tools without using rosbridge by embedding rosbridge application programming interface (API) services.
- Supports user-defined types in ROS interactions with a Python-based dynamic type conversion function.
- Non-invasive design requires no system changes beyond installation of custom ROS message packages.
- Utilizes multiprocessing to address performance degradation caused by Python’s Global Interpreter Lock (GIL).
- Packaged as a Docker container, allowing operation without a direct ROS installation on the host machine.

## 4. Experiments and Results

### 4.1. Experimental Setup

The experiments were conducted on a Robot Node and a Server Node, which were connected via a 100 Mbps network. The experimental setup was configured in a slightly different manner for each scenario, including those designated as *localhost*, *local-subnet*, *Gateway*, and *rosbridge*. A detailed description of these configurations can be found in Section 4.2. In these experiments, ROS topics were configured to publish only from ROS programs on the robot node with a sensor device. Concurrently, performance metrics (e.g., *rate*, *delay*, *message size*) were recorded for all target topics (e.g., RGB image, depth image) on the server node in all scenarios except the *localhost scenarios*. A summary of the experimental setup is provided in Table 4.

The calculation of each measurement metric was performed as follows:

- Rate (Hz): The rate was calculated as the number of messages received per second. This was measured using the command “ros2 topic hz”. The formula for rate is:
  (1) $$R=\frac{N}{\left(\sum_{x=1}^{N}\left(T_{x}-T_{x-1}\right)\right)},$$
  where *R* is the average rate of the observed topics; $T_{x}$ denotes the observed time of the *x*-th published topics; and *N* is the number of observed topics, which is set to a constant value. The initial value, $T_{0}$, was set to the same value as $T_{1}$.
- Delay (seconds): The delay was calculated as the average time taken for a message to be received after it had been sent. This was measured using the command “ros2 topic delay”. The formula for delay is:
  (2) $$D=\frac{\left(\sum_{x=1}^{N}\left(T_{x}-H_{x}\right)\right)}{N},$$
  where *D* is the average delay of the observed topics; $T_{x}$ denotes the observed time of the *x*-th published topics; $H_{x}$ denotes the topic creation time contained in the header of the published topic $T_{x}$; and *N* is the number of observed topics, which was set to a constant value.
- Message size (megabytes): The message size was dependent on the data type of the observed topic and was a constant value for each topic in this experiment.

### 4.2. Experimental Scenarios

#### 4.2.1. Localhost Scenarios

The *localhost* scenarios were designed to assess the baseline performance of ROS on the Robot Node. The sensor ROS program published RGB image and Depth image topics in the Robot Node, and performance metrics were measured using ROS performance measurement tools on the same Robot Node.

#### 4.2.2. Local-Subnet Scenarios

The *local-subnet* scenarios were designed to evaluate the performance of ROS communication between the Robot Node and the Server Node on the same network (i.e., the same subnet). The sensor ROS program, which was executing on the Robot Node, published RGB image and depth image topics. The performance metrics were evaluated using the ROS performance measurement tools on the Server Node.

#### 4.2.3. Gateway Scenarios

The *Gateway* scenario was employed to assess the performance of the Gateway under varying configuration options, as detailed in Table 4. Both the Robot and Server nodes ran the Gateway in separate Docker containers, with each Docker container configured to utilize a separate Docker network. The Gateway on the Robot Node utilized the same Docker network as the Docker container running the sensor ROS program. In the Topic forwarding experiments, the Gateway on the Robot Node was configured to connect to the Gateway on the Server Node, while in the Topic reverse forwarding experiments, the Gateway on the Server Node was configured to connect to the Gateway on the Robot Node. Performance measurements were conducted using a measurement tool on separate ROS containers that were running within the Server Node.

#### 4.2.4. Rosbridge Scenarios

The *rosbridge* scenario was employed to assess the performance of rosbridge for representative options (see Table 4) available in the current version of rosbridge (version 1.3.2 [24]). The Gateway and rosbridge were configured to operate within separate Docker containers on both the Robot Node and the Server Node. Furthermore, each Docker container was configured to utilize a distinct Docker network. The Gateway or rosbridge running on the Robot Node utilized the same Docker network as the Docker container in which the sensor ROS program was running. The topic forwarding experiment was configured so that the Gateway on the Robot Node forwarded topics to rosbridge on the server node. The topic reverse forwarding experiment was configured so that the Gateway on the server node received Topics from rosbridge on the Robot Node. Performance measurements were performed using the measurement tool on a separate ROS container running on the Server Node. In this experiment, the Gateway acted as a client program to rosbridge, which only functions as a server.

### 4.3. Experimental Results

The experimental results were evaluated based on two primary metrics for the observed topics: topic *rate* and topic *delay*. The “Comparative Experimental Results” section (see Section 4.3.1) presents a comparison of the performance of the Gateway and rosbridge under a variety of configuration scenarios. The section “Gateway Performance Experimental Results” (see Section 4.3.2) provides performance measurements for each configuration option provided by the proposed Gateway. The *message size* metric was measured but not plotted on a graph because it has a constant value for each topic and was therefore used in the analysis of the results for each metric.

#### 4.3.1. Comparative Experimental Results

**Figure 3:**
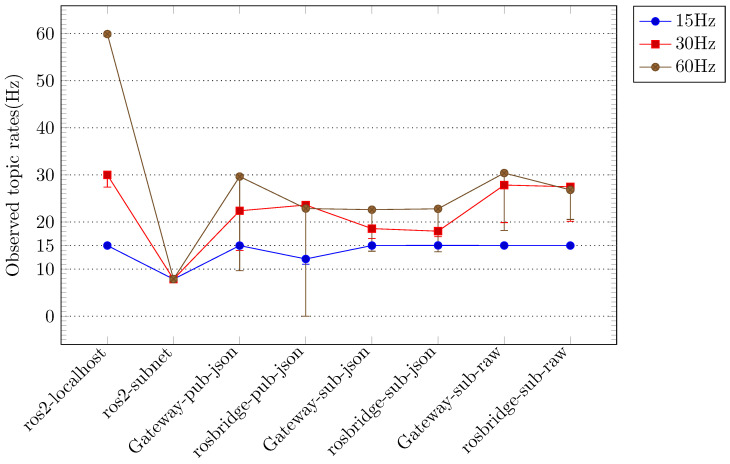
Comparison of ROS topic transmission rates for different configurations: The configurations include the use of ROS on a single device (*ros2-localhost*) and local subnet (*ros2-subnet*), the use of the Gateway on separate networks (*Gateway-pub-json, Gateway-sub-json, Gateway-sub-raw*), and the use of rosbridge on separate networks (*rosbridge-pub-json, rosbridge-sub-json, rosbridge-sub-raw*). The best performance for each configuration is plotted, with error bars representing the worst performance. Higher values indicate superior performance.

**Figure 4:**
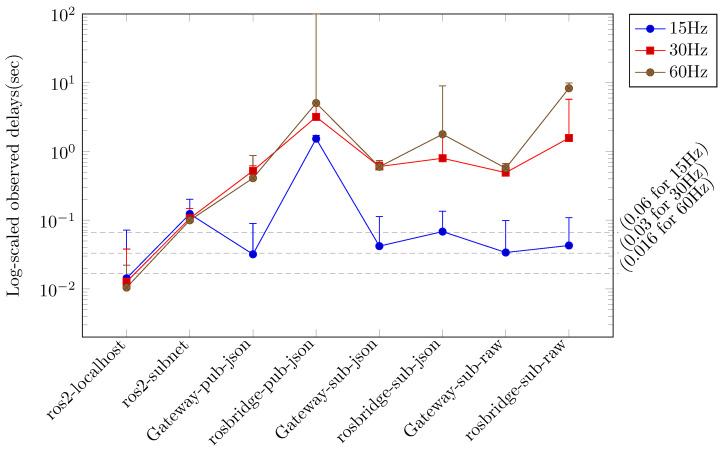
Comparison of ROS topic transmission delay for different configurations: The configurations include the use of ROS on a single device (*ros2-localhost*) and local subnet (*ros2-subnet*), the use of the Gateway on separate networks (*Gateway-pub-json, Gateway-sub-json, Gateway-sub-raw*), and the use of rosbridge on separate networks (*rosbridge-pub-json, rosbridge-sub-json, rosbridge-sub-raw*). A log scale is used to illustrate the delay values. The minimum delay for each configuration is plotted, and the error bars represent the maximum delay. Lower values indicate better performance. The horizontal dashed lines on the graph represent the real-time limit delay at each occurrence rate.

In each scenario, the performance metrics were evaluated at varying sensor data generation rates (15 Hz, 30 Hz, and 60 Hz), and the primary performance metrics of *rate* and *delay* were compared. Figure 3 illustrates the comparative outcomes for the *rate* metric, while Figure 4 depicts the comparative outcomes for the *delay* metric. In Figure 3 and Figure 4, the measurement results are displayed according to the sensor data generation rate and classified according to the applied scenario. Figure 3 depicts the *rate* measurement outcomes for each scenario, primarily illustrating the maximum *rate* when image and depth topics are employed in conjunction. The lowest *rate* observed in each configuration is indicated by the error bars. Figure 4 depicts the *delay* measurement results for each scenario, primarily illustrating the minimum *delay* value when image and depth topics are employed concurrently. The maximum *delay* value in each configuration is indicated by the error bars. In Figure 3 and Figure 4, the measurement results for the localhost scenario using ROS within a single device are indicated as *ros2-localhost*. The results of the measurements conducted for the scenario in which ROS is employed across multiple hosts within a local network are presented as *ros2-subnet*. In scenarios where JSON-encoded topics are forwarded to a remote Gateway or remote rosbridge, the measurement results are designated as *Gateway-pub-json* and *rosbridge-pub-json*, respectively. In scenarios where JSON-encoded topics are reversed from a remote Gateway or remote rosbridge, the measurement results are designated as *Gateway-sub-json* and *rosbridge-sub-json*, respectively. Similarly, in scenarios where raw-encoded topics are reversed from a remote Gateway or remote rosbridge, the measurement results are designated as *Gateway-sub-raw* and *rosbridge-sub-raw*, respectively.

#### 4.3.2. Gateway Performance Experimental Results

**Figure 5:**
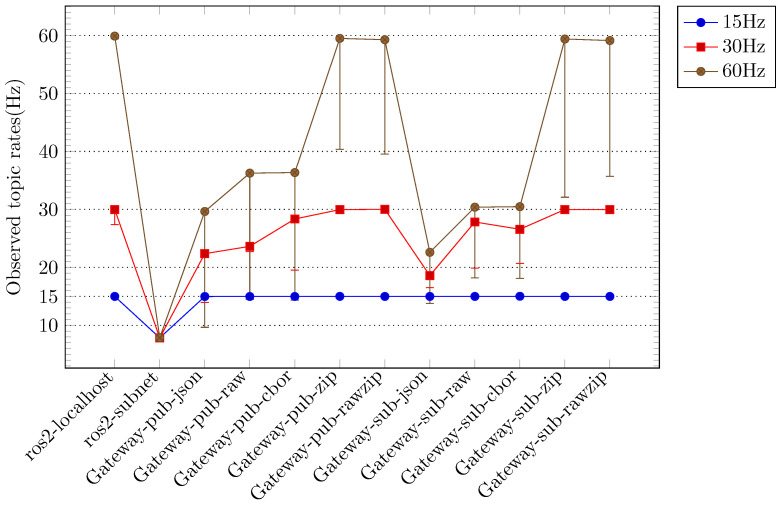
Observed topic rates by Gateway configurations and sensor publishing rates: The configurations include the use of ROS on a single device (*ros2-localhost*) and local subnet (*ros2-subnet*), as well as the use of the Gateway on each option. The best performance for each configuration is plotted, with error bars representing the worst performance. Higher values indicate superior performance.

**Figure 6:**
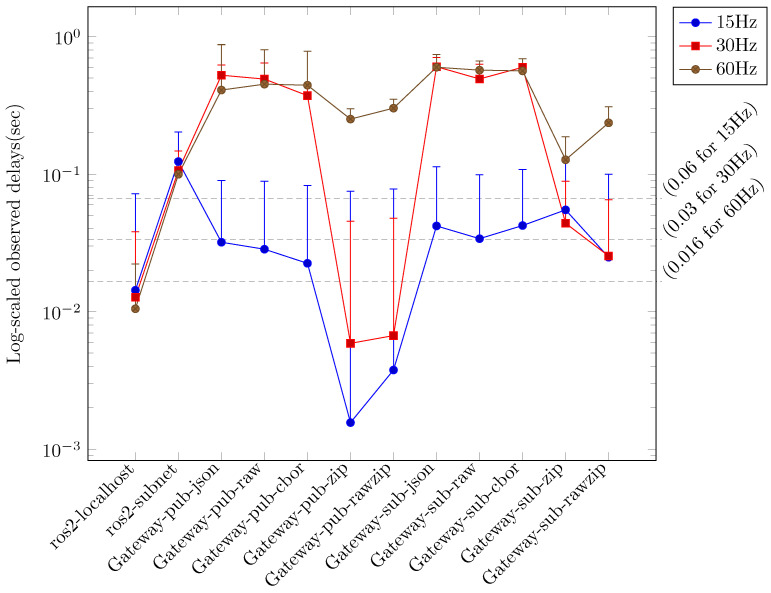
Observed topic delays by Gateway configurations and sensor publishing rates: The configurations include the use of ROS on a single device (*ros2-localhost*) and local subnet (*ros2-subnet*), as well as the use of the Gateway on each option. A log scale is used to illustrate the delay values. The minimum delay for each configuration is plotted, and the error bars represent the maximum delay. Lower values indicate better performance. The horizontal dashed lines on the graph represent the real-time limit delay at each occurrence rate.

A series of experiments was conducted to compare the performance of the Gateway options when the sensor data generation rate was altered to 15 Hz, 30 Hz, and 60 Hz. All available Gateway options were employed in both topic forwarding and topic reverse forwarding operations. Figure 5 illustrates the outcomes of the *rate* metric for all options within the Gateway scenario, whereas Figure 6 depicts the results of the *delay* metric. In Figure 5 and Figure 6, the measurements are displayed according to the sensor data generation rate and sorted by the applied options. For purposes of comparison with pure ROS, Figure 5 and Figure 6 present the *ros2-localhost* and *ros2-subnet* measurements together. In Figure 5 and Figure 6, each result of the Gateway experiment is labeled in the format “Gateway-*task-option*”. In regard to the *task* directive of label, the term “pub” is used to denote the results of the topic forwarding operation, whereas the term “sub” is used to indicate the results of the topic reverse forwarding operation. The *option* directive reflects the designation of the option provided by the Gateway (see Section 3.2), with the exception of the *cbor-raw* option, which is labeled as *raw*.

### 4.4. Discussion

As anticipated, the *localhost* scenarios with no network transport exhibited the highest *rate* and lowest *delay*. Figure 3 illustrates that the *local-subnet* scenarios exhibited the poorest rate measurement results for all data generation rates. The results of these experiments using ROS were found to be attributable to the network bandwidth required, in accordance with the network connection characteristics of the ROS topic task. The following equations represent the calculation of the network bandwidth required for a ROS application comprising host set *H* and topic set *T*, along with the expected subscribing topic rate when the network bandwidth is specified.

(3) $$B=\sum_{i\in T}\sum_{\begin{array}{c} j\in H\\ j^{\prime }\in H\\ j\neq j^{\prime } \end{array}}\left(S\left(i\right)\cdot Rp\left(i\right)\cdot Np\left(i,j\right)\cdot Ns\left(i,j^{\prime }\right)\cdot nbits\right),$$

(4) $$Rr\left(i\right)=\frac{Rp(i)\cdot L}{B},$$

where S(i) is the message size of topic *i*, Rp(i) is the publishing rate of topic *i*, Np(i,j) represents the number of publishers for topic *i* on host *j*, $Ns(i,j^{\prime })$ represents the number of subscribers for topic *i* on host $j^{\prime }$, *L* represents the given inter-host network bandwidth, Rr(i) is the predicted subscribing rate for topic *i*, and nbits represents the conversion factor from bytes to bits.

The *Gateway* and *rosbridge* scenarios demonstrated disparate performance measurements contingent on the configuration options. In particular, Figure 3 indicates that the *raw* results for the *Gateway* and *rosbridge* scenarios exhibited the most favorable performance when compared to the *json* results. Furthermore, the difference between the *raw* results for the two scenarios was relatively minimal. The *Gateway* scenarios exhibited an average rate that was approximately 1.5 times higher than the *rosbridge* scenarios in the topic forwarding task with the *json* option. The low delay measurement result for the *local-subnet* scenarios in Figure 4 can be attributed to the low rate. At a low sensor data generation rate of 15 Hz, the topic forwarding task with the *json* option in the *rosbridge* scenarios exhibited superior rate measurement results compared to the *local-subnet* scenarios. However, the delay measurement results were the most unfavorable compared to all other scenarios. The *Gateway* scenarios exhibited delay measurement results that were, on average, more than seven times superior to those of the *rosbridge* scenarios at both high sensor data generation rates (30 Hz and 60 Hz).

The following equation calculates the expected network bandwidth when the Gateway $B_{g}$ is applied to a ROS application consisting of a set of hosts *H* and a set of topics *T*:

(5) $$B_{g}=\frac{1}{Co}\sum_{i\in T}\sum_{\begin{array}{c} j\in H\\ j^{\prime }\in H\\ j\neq j^{\prime } \end{array}}\left(S\left(i\right)\cdot Rp\left(i\right)\cdot Ep\left(i,j\right)\cdot Es\left(i,j^{\prime }\right)\cdot nbits\right),$$

where S(i) denotes the message size of topic *i*, Rp(i) represents the publishing rate of topic *i*, Ep(i,j) is a Boolean indicating whether host *j* publishes topic *i* and $Es(i,j^{\prime })$ is a Boolean indicating whether host $j^{\prime }$ subscribes to topic *i*, nbits represents the conversion factor from bytes to bit (typically 8), and Co represents the compression ratio specific to the Gateway’s options. For the *zip* and *rawzip* options, the default compression ratio [51] of “lz4” is applied.

Figure 5 and Figure 6 illustrate that the results for the *Gateway* scenarios at sensor data rates of 15 Hz and 30 Hz are in close alignment with the ideal rates observed in the *localhost* scenarios. The *delay* metric results for the *Gateway* scenarios at a sensor data rate of 15 Hz, particularly when the *zip* and *rawzip* options were applied to topic forwarding tasks, exhibited a deviation of less than 5% from the ideal delay values observed in the *localhost* scenarios, which were deemed acceptable. However, in the 30 Hz experiment, the delays exceeded the ideal values by more than 20%, indicating a deviation from the desired outcome. Figure 5 and Figure 6 also illustrate that tasks utilizing the *zip* and *rawzip* options in the *Gateway* scenarios, with the exception of the 60 Hz experiment, exhibited superior performance in both the *rate* and *delay* metrics in comparison to the *local-subnet* scenarios. In the 60 Hz experiments, the *Gateway* scenario with *zip* and *rawzip* options achieved notable *rate* results, reaching 75% of the ideal *rate* in the *localhost* scenarios, despite the total amount of topics to be transferred exceeding the 100 Mbps network bandwidth. The *delay* metrics in these experiments exceeded the 60 Hz sensor data generation period of 0.016 s, which may be a cause for concern. However, it is noteworthy that more than 40 sensor data points were still collected per second. In summary, these experiments demonstrate the significant impact of configuration choices on system performance in cloud robotics environments. Specifically, Gateway configurations that leverage compression options show great potential for improving both data transfer efficiency and delay management under a variety of operating conditions.

Further inquiries pertaining to the experiments revealed the following factors that contributed to each outcome:

- The inferior performance of rosbridge: Experimental logs confirmed that rosbridge was unable to receive binary data, limiting its ability to handle large data. Its asynchronous implementation also caused CPU performance issues due to Python’s GIL, which blocked multi-topic data reception.
- Decline in performance of applications on local subnets relying solely on ROS: In the performance experiment, executing commands for three subscribers per topic resulted in a threefold increase in transmission due to ROS’s peer-to-peer connection. This issue can also arise when different applications on the same host utilize the same topic from a different host.

Furthermore, we considered potential limitations or edge cases where the Gateway may not significantly enhance ROS availability. These include:

- Gateway topic forwarding when ROS peers are unavailable: ROS only transmits data when a peer is present. However, since the Gateway acts as a peer, it consumes bandwidth even in the absence of an actual ROS peer, which could negatively affect the transmission performance of other key topics.
- Applications on the same machine using distinct topics within a single subnet: If multiple applications on the same machine are not repetitively listening to the same topics, using a Gateway may introduce more overhead compared to using ROS alone.
- Time-sensitive application environments: The Gateway is not designed to address time-critical problems, so it does not improve ROS availability in environments where strict timing constraints are crucial.

## 5. Conclusions

In this paper, we introduce the ROS Gateway, a middleware solution designed to facilitate seamless collaboration between a robot’s onboard sensors and cloud-based AI systems. We provide a comprehensive account of the architectural, protocol, and algorithmic aspects of the ROS Gateway, along with an illustration of how it facilitates more efficient communication between distributed systems. The experimental results demonstrate that the ROS Gateway enhances performance across a range of configurations, particularly in cloud and fog robotics environments.

The study yielded several key findings, which are outlined below:

- The ROS Gateway has the potential to outperform a standard ROS-only configuration, even when the robot and server are on the same local subnet, depending on the distribution of ROS applications.
- The transmission of sensor data from advanced devices, such as depth cameras, was more efficient and reliable with the ROS Gateway, thereby reducing delays in processing critical environmental information and improving the robot’s overall responsiveness.
- The formulas provided for predicting topic reception rates in both ROS and the ROS Gateway offer valuable guidance for the design of optimal deployment strategies for sensor-rich robotic systems, particularly in complex and data-intensive environments.

These findings highlight the potential of the ROS Gateway to significantly enhance the performance of robotic systems in distributed computing environments, making it a valuable tool for developers seeking to optimize robotic applications.

In future work, we intend to investigate the influence of security measures on system performance, examine the potential of techniques such as NAT Traversal, and optimize the system for resource-constrained devices through the use of low-level programming languages. By addressing these factors, our objective is to enhance the robustness and efficiency of sensor data processing in the cloud and fog robotics, and ensure reliable operation even in resource-constrained scenarios.

## Figures and Tables

**Table 1:** Challenges for cloud robotics middleware.

Challenges	Description
No modification of ROS-based applications or the ROS framework	Modifying the framework would require recompilation and redistribution of the entire application, which can be time-consuming and inefficient.
Operation of robots behind Network Address Translation (NAT) or firewalls	Mobile robots often lack static IP addresses and operate in environments where firewalls and NAT are present.
Compatibility with web-based ROS tools	Many robotics projects depend on web-based ROS tools such as Foxglove Studio [38], roslibjs [39], and roslibpy [40].
Use of cloud-friendly protocols	Web protocols are essential for optimizing scalability and performance on cloud platforms.

**Table 2:** Comparison of the proposed work with previous studies.

Feature	Rosbridge Based	VPN Based	ZeroMQ Based	MQTT Based	Out Work
	[25,26,27]	[29,30,31]	[28]	[34,35,36]	
ROS Topic	Yes	Yes	No	Yes	Yes
ROS Service	Yes	Yes	No	No	Yes
ROS Action	Yes	Yes	No	No	Yes
Provisioning	Yes	Yes	Yes	Yes	Yes
Cloud to Robot	No	No	Yes	Yes	Yes
Non-invasive	No *	No ^†^	No	No	Yes
General Purpose	No *	Yes	No	No	Yes
NAT/Firewall	No	Yes	Yes	Yes	Yes
Web Compatibility	Yes	No	No	No	Yes
Cloud Protocol	Yes	No	No	Yes	Yes
Multi-Host ROS	Yes	No	No	No	Yes
Large Data	No	No	Yes	Yes	Yes

* These systems do not function as standalone entities; rather, they require the presence of application-specific clients for their operation. ^†^ For the systems to function, they must be installed on every host with which they interact.

**Table 3:** Gateway protocol message specification.

Message Name	Specification	Description
(a) AdvertiseTopicMsg	{“op”:“advertise”, *(optional)*“id”:*<string>*, “topic”:*<string>*, “type”:*<string>*, (*optional* *)“compression”:*<string>*}	Notifies the recipient about a topic that will be forwarded.
(b) PublishTopicMsg	{“op”:“publish”, *(optional)*“id”:*<string>*, “topic”:*<string>*, “msg”:<json> or <bytes *> }	Sends ROS topic data for forwarding or reverse forwarding.
(c) SubscribeTopicMsg	{“op”:“subscribe”, (*optional*)“id”:*<string>*, “topic”:*<string>*, *(optional)*“type”:*<string>*, *(optional ^†^)*“compression”:*<string>*}	Requests the Gateway to subscribe to a ROS topic for reverse forwarding.
(d) AdvertiseServiceMsg	{“op”:“advertise_service”, “type”:*<string>*, “service”:*<string>*, (*optional* ^‡^)“compression”:*<string>*}	Informs Gateways about the availability of a ROS service.
(e) CallServiceMsg	{“op”:“call_service”, (*optional*)“id”:*<string>*, “service”:*<string>*, (*optional*)“args”:list<json>> or <bytes *>, *(optional ^‡^)*“compression”:*<string>*}	Sends service requests to Gateways in different networks.
(f) ServiceResponseMsg	{“op”:“service_response”, (*optional*)“id”:<string>, “service”:*<string>*, (*optional*)“values”:<json> or <bytes *>, “result”:*<boolean>*}	Sends service results to the requesting Gateway.
(g) AdvertiseActionMsg	{“op”:“advertise_action”, “type”:*<string>*, “action”:*<string>*, (*optional* *)“compression”:<string>}	Announces the availability of a ROS action.
(h) SendActionGoalMsg	{“op”:“send_action_goal”, (*optional*)“id”:*<string>*, “action”:*<string>*, “action_type”:*<string>*, (*optional*)“args”:*<json>*, (*optional*)“feedback”:*<boolean>*, (*optional* ^‡^)“compression”:*<string>*}	Sends the action goal to the advertising Gateway.
(i) ActionFeedbackMsg	{“op”:“action_feedback”, “id”:*<string>*, “action”:*<string>*, “values”:<json> or <bytes *>}	Transmits feedback during action processing.
(j) ActionResultMsg	{“op”:“action_result”, “id”:*<string>*, “action”:*<string>*, “values”:<json> or <bytes *>, “result”:*<boolean>*}	Sends the result of a ROS action to the requester.

* Features added to the existing rosbridge protocol. ^†^ In the rosbridge protocol, the values can be none, png, cbor, and cbor-raw, but in this study, zip and rawzip have been added instead of png. ^‡^ The rosbridge protocol permits the use of the values none and png, whereas this study permits the use of the values cbor, cbor-raw, zip, and rawzip instead of png.

**Table 4:** System configuration.

Configuration	Details
Hardware Configuration	
Robot Node	
Processor and memory	ARM Cortex-A78AE 2.2 GHz, 64 GB LPDDR5 RAM (NVIDIA Jetson AGX Orin [46]) (Santa Clara, CA, USA)
Additional devices	Intel RealSense Camera (Santa Clara, CA, USA) [47]
Server Node (Emulate Cloud or Fog server)	
Processor and memory	AMD Ryzen 9 3900X (Santa Clara, CA, USA), 64 GB DDR4 RAM
Software Environment	
Host operating system	Ubuntu 20.04 LTS (server, robots)
Middleware platform	ROS 2 Foxy with DDS(Fast RTPS) [48]
Docker version	20.10.7
Containers	Separate containers for each ROS node, rosbridge and Gateway
Container OS	Ubuntu 20.04 LTS (docker Images)
Programming language	Python 3.8
Measurement tools	ros2cli [49] (“ros2 topic hz”, “ros2 topic delay”, “ros2 topic bw”)
Measurement metrics	rate (Hz), delay (sec), message size (megabytes)
Network Configuration	
Network bandwidth	100 Mbps
Experiments Configuration	
Publisher	Realsense ROS node [50]
Topics published	424 × 240 × 3 RGB Image (message size: 0.31 Mbytes), 424 × 240 × 2 Depth Image (message size: 0.2 Mbytes)
Publishing rates	15 Hz, 30 Hz, 60 Hz
Target tasks	Topic tasks (forwarding, reverse forwarding)
Gateway options	cbor-raw, json, cbor, zip, rawzip (for all Topic tasks)
Rosbridge options	cbor-raw (for only topic reverse forwarding), json (for all topic tasks)

## Data Availability

The source-code of the ROS Gateway is openly accessible in the GitHub repository: https://github.com/parasby/rosgw (accessed on 14 July 2024).

## Abbreviations

The following abbreviations are used in this manuscript:

AI	Artificial Intelligence
IOT	Internet of Things
ROS	Robot Operating Systems
LAN	Local Area Network
IP	Internet Protocol
JSON	JavaScript Object Notation
PC	Personal Computer
NAT	Network Address Translation
AWS	Amazon Web Services
UAV	Unmanned Aerial Vehicle
VPN	Virtual Private Network
MQTT	Message Queuing Telemetry Transport
C-ITS	Cooperative-Intelligent Transport Systems
EDRP	Edge-Driving Robotics Platform
LDMP	Local Dynamic Map Platform
5G	5th Generation mobile communications
PNG	Portable Network Graphics
CBOR	Concise Binary Object Representation
URI	Uniform Resource Identifier
API	Application Programming Interface
GIL	Global Interpreter Lock
Mbps	megabits per second
DDS	Data Distribution Service
RTPS	Real Time Publish/Subscribe
RGB	Red, Green, Blue
Hz	Hertz
Gateway	The ROS Gateway

## Appendix A. Gateway Task Sequence Diagram

The Gateway initiates with the BringUp Task Algorithm 1 and subsequently diverges into the Topic Task Algorithm 2, Service Task Algorithm 3, and Action Task Algorithm 4, based on the configuration parameters. Each task operates as a discrete process for its respective configuration item, and these processes collaborate by exchanging messages between Gateways, thereby enhancing ROS’s availability. In the figures, “Subnet L” is used to refer to the robot’s internal network, or the local network to which the robot is connected. In contrast, “Subnet R” represents the network that is connected to external servers, such as remote clouds and fog nodes. In the majority of cases, Subnet L is assigned a private IP address, whereas Subnet R is allocated a public IP address.

Figure A1 depicts the process of forwarding topics published by the robot to the cloud (topic forwarding) and retrieving topics from the cloud by the robot (topic reverse forwarding). The ROS protocol serves to facilitate communication between the ROS node and the Gateway.

Figure A2 illustrates the process of exposing the robot’s ROS services to the cloud (Expose Service) and reserving ROS services hosted in the cloud for the robot’s use when needed (Reserve Service). The Gateway provides a proxy server function, thereby enabling it to virtually implement a ROS service and receive service calls from ROS nodes. An alternative option is for it to act as a proxy client, processing ROS service requests from other Gateways.

Figure A3 illustrates the process of exposing the robot’s ROS actions for use in the cloud (Expose Action) and reserving ROS actions running in the cloud for the robot’s use when needed (Reserve Action). This process is similar to the Service Task, but involves a more complex algorithm to manage the delivery of action feedback messages from the ROS node.
